# Selfish elements: genetics of natural and synthetic systems

**DOI:** 10.1093/g3journal/jkaf167

**Published:** 2025-08-06

The journals of the Genetics Society of America, GENETICS and G3: Genes|Genomes|Genetics, are calling for submissions of papers on selfish genetic elements.

Selfish elements bias their own transmission to the next generation, often at an expense to the host that harbors them. Once considered oddities, it is now clear that selfish elements take many forms and are widespread across the tree of life. Early studies laid the foundation for the presence and evolutionary dynamics of selfish elements, and modern investigations show their far-reaching consequences for molecular processes and genome evolution. In complement, based on an understanding of naturally occurring selfish element mechanisms and dynamics, synthetic genetic elements are being developed to suppress or alter traits of pest species.

This series will highlight the genetic mechanisms and evolutionary consequences of selfish non-Mendelian inheritance. Specifically, we invite high-quality submissions with a focus on selfish non-Mendelian transmission, including both theoretical and empirical studies of natural and synthetic systems. This series welcomes studies on meiotic drive, segregation distortion, synthetic drive, *Wolbachia* and other reproductive manipulators, B-chromosomes, toxin-antidote systems, and quirky inheritance systems.

**Series Editors:**

- Kelly Dyer, University of Georgia, USA
- Fred Gould, North Carolina State University, USA
- Amanda Larrracuente, University of Rochester, USA

The Series will launch in Fall 2026, though articles will be published online shortly after acceptance via Advance Access. Papers will be collected and navigable across the two journals and promoted on social media and other venues in a manner similar to other series the journals have published.

Authors are invited to submit manuscripts by October 1, 2025, and should submit to their journal of choice. Manuscripts will be reviewed and edited through the standard peer review process and according to the usual high standards of the journals. Some manuscripts submitted to GENETICS may be offered a transfer to G3: Genes|Genomes|Genetics instead.

We encourage continued submissions past October 1, 2025 as we intend this series to be an ongoing and growing resource in this area. At submission, please choose the “Selfish elements: genetics of natural and synthetic systems” article type in the submission system and mention the series in your cover letter.

Please share with your colleagues, and do not hesitate to contact any of the series editors or the GENETICS and G3: Genes|Genomes|Genetics Editorial Office with questions, suggestions, or presubmission inquiries.

